# Bromoenol Lactone Attenuates Nicotine-Induced Breast Cancer Cell Proliferation and Migration

**DOI:** 10.1371/journal.pone.0143277

**Published:** 2015-11-20

**Authors:** Lindsay E. Calderon, Shu Liu, Nova Arnold, Bethany Breakall, Joseph Rollins, Margaret Ndinguri

**Affiliations:** 1 Department of Biology, Eastern Kentucky University, Richmond, KY, 40475, United States of America; 2 Department of Physiology, Hebei University Health Science Center, Hebei, 071000, China; 3 Department of Chemistry, Eastern Kentucky University, Richmond, KY, 40475, United States of America; Stony Brook University, UNITED STATES

## Abstract

**Objectives:**

Calcium independent group VIA phospholipase A_2_ (iPLA_2_β) and Matrix Metalloproteinase-9 (MMP-9) are upregulated in many disease states; their involvement with cancer cell migration has been a recent subject for study. Further, the molecular mechanisms mediating nicotine-induced breast cancer cell progression have not been fully investigated. This study aims to investigate whether iPLA_2_β mediates nicotine-induced breast cancer cell proliferation and migration through both *in-vitro* and *in-vivo* techniques. Subsequently, the ability of Bromoenol Lactone (BEL) to attenuate the severity of nicotine-induced breast cancer was examined.

**Method and Results:**

We found that BEL significantly attenuated both basal and nicotine-induced 4T1 breast cancer cell proliferation, via an MTT proliferation assay. Breast cancer cell migration was examined by both a scratch and transwell assay, in which, BEL was found to significantly decrease both basal and nicotine-induced migration. Additionally, nicotine-induced MMP-9 expression was found to be mediated in an iPLA_2_β dependent manner. These results suggest that iPLA_2_β plays a critical role in mediating both basal and nicotine-induced breast cancer cell proliferation and migration *in-vitro*. In an *in-vivo* mouse breast cancer model, BEL treatment was found to significantly reduce both basal (p<0.05) and nicotine-induced tumor growth (p<0.01). Immunohistochemical analysis showed BEL decreased nicotine-induced MMP-9, HIF-1alpha, and CD31 tumor tissue expression. Subsequently, BEL was observed to reduce nicotine-induced lung metastasis.

**Conclusion:**

The present study indicates that nicotine-induced migration is mediated by MMP-9 production in an iPLA_2_β dependent manner. Our data suggests that BEL is a possible chemotherapeutic agent as it was found to reduce both nicotine-induced breast cancer tumor growth and lung metastasis.

## Introduction

According to the American Cancer Society, Cancer *Facts & Figures 2015*, there are an estimated 1,658,370 new cancer cases diagnosed in the United States, of which 231,840 are attributed to breast cancer with 40,290 deaths. Breast carcinomas are at the forefront of cancer cases among women (41%) followed by uterine corpus (8%), and colon and rectum cancer (8%) [1]. Additionally, in the United States breast cancer afflicts 1 in 8 women during their lifetime; this high prevalence provides evidence for the need to foster therapeutic interventional research [2].

Of utmost concern is the malignant characteristics of breast cancer, in which, the major cause of mortality in breast cancer patients is metastasis to distant sites rather than the growth of the primary tumor [3]. 10–15% of breast cancer patients develop distant metastases within 3 years after the identification of a primary tumor, and remain at risk for metastatic disease during their entire lifespan [4]. Metastasis is a multi-step process involving local tumor cell invasion, intravasation, survival in the vasculature, extravasation, and colonization at a distant secondary site; all of which are consequently reliant on mechanisms of cell migration and movement [5].

A major risk factor for the development and exacerbation of breast cancer metastasis is cigarette smoke [6,7]. Smoke exposure has been found to increase anchorage-independent growth, motility, and invasiveness of breast cancer cells leading to higher rates of cell survival, colonization [8] and metastasis. Moreover, nicotine a known component in cigarette smoke has been shown to promote cancer development, cancer cell migration, and more recently apoptotic resistance in breast cancer cells [9,10,11]. Additionally, several epidemiological studies have indicated a strong link between smoke exposure and an increased risk of breast cancer [12,13,14]. Specifically, cigarette smoke was found to significantly increase breast cancer mortality by 60% and the probability of recurrence by 41% [15]. Supporting this association is the presence of tobacco carcinogens in the breast tissue of smokers [16]. In particular, nicotine is a known toxicant found in smoke that is linked to breast cancer apoptosis resistance, tumor growth, and migration, along with, facilitating angiogenic metastasis [9,17,18,19,20]. The exact cellular mechanisms promoting nicotine-induced breast cancer cell proliferation and migration remain undetermined.

In recent years, the enzyme iPLA_2_β (calcium-independent phospholipase A2 Beta) has emerged as an important cell membrane regulator and a contributor to various types of cancers including: survival signaling in prostate cancer, along with, proliferation, migration and tumorigenicity in ovarian cancer [21,22,23,24]. iPLA_2_β is a member of the PLA_2_ superfamily and is found both in the cytosol and bound to membranes; upon activation it translocates to the membrane in a calcium-independent process [25]. The other PLA_2_ family members are secretory PLA_2_ (sPLA_2_) and cytosolic (cPLA_2_), which are characteristically different based on their cellular locations and their calcium requirement for enzymatic activation. The primary catalytic function of iPLA_2_β is to cleave glycerophospholipids at the *sn*-2 position to release free fatty acids including arachidonic acid and 2-lysophospholipid [26]. Early on because of its catalytic activity, iPLA_2_β was classified as a housekeeping protein solely responsible for dynamically regulating membrane homeostasis/remodeling by altering the phospholipid composition of the membrane [27,28]. Currently, studies have now implicated iPLA_2_β as an important signaling molecule mediating various cellular functions and the progression of many disease states including hypertension, diabetes, alzheimer’s, and aneurysm formation [29,30,31,32]. Sequentially, iPLA_2_β has been found to regulate numerous membrane functions including vacuole formation, membrane transport, surface adhesion, cellular spreading, and proliferation [33,34].

Current research indicates Bromoenol Lactone (BEL), a suicide inhibitor of iPLA_2_β attenuates cigarette smoke induced-breast cancer cell mobility [7]. However, whether iPLA_2_β is involved in breast cancer tumor growth and metastasis remain unreported. BEL, irreversibly inhibits the enzymatic activity of iPLA_2_β by covalently binding to the lipase motif, rendering it inactive. The sPLA_2_s and cPLA_2_α isoforms, contain an enzymatic lipase domain similar to iPLA_2_; however, BEL has been shown to specifically inhibit iPLA_2_β [35]. Additionally, BEL has been indicated to have a 1000-fold more specificity towards iPLA_2_ than cPLA_2_ [36].

Our *in-vitro* and *in-vivo* studies introduce the novel idea that nicotine from cigarette smoke could enhance iPLA_2_β expression in breast cancer cells leading to enhanced tumor growth, along with, migration and metastatic ability. Here we present the effects of nicotine on cell proliferation and mobility in the 4T1 breast cancer cell line. The 4T1 is a mouse stage IV breast cancer cell line which is transplantable, highly tumorigenic and invasive, and has been characterized to spontaneously metastasize from the primary tumor in the mammary gland to multiple distant sites, imitating human clinical disease [37]. Thus far, the involvement of iPLA_2_β in mediating nicotine-induced breast cancer tumor growth and metastasis remains relatively unstudied and the exact signaling mechanisms regulated by iPLA_2_β in breast cancer could provide a critically needed new target for therapeutic intervention.

## Methods

### Cell Culture

The 4T1 mouse mammary tumor cell line (original commercially obtained from ATCC) was a gracious gift from Dr. Shu Liu and Dr. Kai Su from and stably transfected with GFP (Green Fluorescent Protein). The 4T1 cell line was cultured in Dulbecco’s Modified Eagle Medium (DMEM) supplemented with 10% (vol/vol) Fetal Bovine Serum, 100U/ml penicillin, and 100μg/ml streptomycin. Cultures were maintained in a 37°C tissue culture incubator with a humidified atmosphere of 95% air and 5% CO_2_.

### Animals

Female BALB/c mice, 10 weeks old, were purchased from Jackson Laboratory (BarHarbor, ME). The animals were allowed to acclimate for 1 week before experimentation and were maintained on a 12 hr light and dark cycle, and fed standard rodent chow (Prolab ISOPRO RMH 3000 Irradiated Lab Diet; Purina Mills International). Animal protocols were approved by the committee on animal research care and use at Eastern Kentucky University.

### Nicotine Treatment *In-Vivo* and *In-Vitro*

An average cigarette contains 10-14mg of nicotine and approximately 1–1.5mg of nicotine is absorbed systemically during smoking [38]. Daily exposure to nicotine in cigarette smokers was found to range from 14.9 to 35.4 mg per day [39]. With rapid absorption and distribution in the body once the smoke reaches the alveolar lining, average human smokers have a peak plasma nicotine levels of 10–50 mg/ml (~60–310 nM). Thus, the nicotine levels of (5mg/kg/day) used in our study are well within the range of the plasma of human smokers. For comparison, mice treated with nicotine 13mg/kg/day were found to have plasma nicotine levels of ~49ng/ml or ~300nM [40]. Numerous studies have used a similar range of nicotine in-vivo treatment in mouse models [41,42,43,44]. Subsequently, human autopsy samples from human smokers indicated a high affinity for nicotine in body tissues in which skeletal muscle was found to have comparable nicotine levels to whole blood and distribution to the liver, kidney, spleen and lung where found to be high [38]. Additionally, nicotine is found to accumulate in breast milk of female smokers [12,38]. Moreover, for the in-vitro experiments 10μM concentration of nicotine was added to the cell cultures, similar to other studies [41,45].

### *In Vivo* Xenograft Model of Breast Cancer and Drug Delivery

4T1-GFP cells (1X10^5^) were suspended in 100μl of DMEM not supplemented with FBS and injected into the right second mammary fat pad of female BALB/c mice as previously described in literature [37]. Mini-osmotic pumps (Alzet model 2004, 28-day release, Alza Co., Palo Alto,CA) containing either Nicotine (Sigma-Aldrich [5mg/kg/day]) or Saline (50% DMSO) were subcutaneously implanted on the right flank via an incision in the scapular region. During the procedure the mice were anesthetized by inhalation of isoflurane mixed with O_2_ (3–5% isoflurane/97% O_2_) and maintained by inhalation of isoflurane missed with O_2_ (1–2% isoflurane/97% O_2_) throughout the procedure using a Drager 19.1 model isoflurane machine (Highland Medical Equipment). Daily administration of Bromoenol Lactone (BEL), [Cayman Chemical Company, lot 70700; (10ug/g/day)] or saline was delivered by intraperitoneal injection throughout the experiment. Treatment administration and scheduling during tumor growth was concurrent with previous studies [46,47,48].

Tumor growth was monitored daily and tumor volumes (mm^3^) were calculated using the formula: (width)^2^ × length/2, where width is the smaller of the two measurements. At 2 weeks the mice were sacrificed and tumor volume and weight were measured. Isolated tissues were either formalin fixed or mounted in Optimal Cutting Temperature compound (OCT; Tissue-Tek, Torrance, CA).

### Histological and Immunohistochemical Staining

Additionally, paraffin-embedded sections (5μm thick) were deparaffinized using xylene, rehydrated, and immunohistochemistry conducted as previously described [49]. Slides were incubated with the following primary antibodies overnight at 4°C antibodies iPLA_2_β (1:100, gracious gift from Dr. Guo University of Kentucky, MMP-9 (1:500, Cell Signaling), MMP-2 (1:500, IHC World), GFP (1:500, Cell Signaling). Followed by counterstaining with hematoxylin and mounted on slides with paramount. Additionally, lung and liver sections were stained with Hematoxylin & Eosin (Surgipath). Representative images were taken under 20 to 40x magnification by Olympus IX70 microscope equipped with Olympus DP70 digital camera. Lung tumor area was analyzed using an Olympus digital camera with Olympus MicroSuit-B3 Software

Tissue mounted in Optimal Cutting Temperature compound (OCT; Tissue-Tek, Torrance, CA) was sectioned (5-μm), incubated in 4% paraformaldehyde, and blocked with normal goat serum (Vectastain ABC kit). Slides were incubated with CD31 antibody (1:200,Abcam) and counterstained with DAPI and mounted with glycerol gelatin (Sigma). Images were taken with an Olympus IX70 microscope equipped with Olympus DP70 digital camera.

### Cell Proliferation

To determine the lowest nicotine concentration to significantly induce cell proliferation a cell count assay was conducted. Cells were seeded 1x10^4^ in 6 well plates in media containing 10%FBS and treated with DMSO (control) or Nicotine (1uM, 5μM, or 10μM). Specifically, S-BEL (Cayman Chemical Company, 10006801) was used for all *in-vitro* experiments. Cells were allowed to expand for 24, 48, and 72 hours. To assess cell proliferation, the cells were trypsinized, suspended with typhan blue, and viable cells counted using a hemocytometer.

To assess the ability of BEL to attenuate nicotine-induced proliferation a MTT Assay was utilized. Cells were seeded 500 cells/100μl in a 96-well plate. The wells were treated with DMSO (control), 10μm Nicotine, 3μm BEL or both BEL and Nicotine; with retreatment after 24 hours. After 48 hours, cells were incubated at 37°C for 4 hours in 10μl MTT Solution obtained from Vbrant MTT Cell proliferation assay kit (Life Technologies). Cells were solubilized and mixed with SDS (sodium dodecyl sulfate). And the absorbance read at 595 nm on a Phenix Genios Tecon 96 well plate reader.

### Cell Migration

Cell migration was examined using a scratch/wound healing assay and a transwell assay. For the scratch assay, cells were cultured in a 6-well plate until a confluent monolayer was formed. A 20μl pipet tip was used to scratch the wells. The wells were sequentially rinsed with PBS and cultured in DMEM supplemented with 10% FBS. The wells were treated with DMSO (control), 10μm Nicotine, 3μm BEL or both BEL and Nicotine. Four representative 10x images were taken at 0, 6, and 24 hours and the gap width was quantified using an average of three leading edge measurements for each image.

To further cell migration a transwell assay was conducted. For the transwell assay, 1X10^5^ cells were treated with 3μm BEL (30 minutes pretreatment), 10μm Nicotine, both BEL and Nicotine, or DMSO (control). The cells were seeded in the upper insert (8-μm pore; corning) using DMEM containing 0.1% FBS and the fitted culture dishes contained 10% FBS. After 4 hours the chambers were removed from the plates, fixed, and the migrated cells were DAPI stained. For each well four representative 20x images were quantified.

### Gel Zymography

To identify which MMP (matrix metalloproteinase) is the major player secreted by the Nicotine stimulation, a serial of parallel gel zymography experiments were performed. The gelatin zymography experiments were conducted without serum in the culture medium; all the treatments occurred 24 hours after starvation. There were no MMPs in the culture medium. The cells (1x10^6^) were treated as described above for 24 hours and 10μl of culture medium was tested. The samples were ran with native Tris-Glycine containing 0.15% gelatin, incubated with reaction buffer, and stained with GelCode Blue Phalloidin (Life Technologies, Grand Island, NY). The bands were scanned and band density analyzed with ImageJ software.

### *In situ* Zymography

To investigate nicotine-induced MMP-9 production 4T1 cells were seeded (1X10^4^) on cover slides, serum starved for 24 hours then treated for 24 hours as mentioned above. The procedure was conducted as previously described [49] with cells incubated with MMP-9 (1:500, Cell Signaling) and counterstained by DAPI.

### Statistics

Data were pooled prior to analyses. Data are illustrated as mean ± SEM and statistical analyses were carried out using GraphPad, Prism 6 (San Diego, CA). T-tests along with one- and two-way ANOVAs were used where appropriate. For study data used in the statistical analysis and figures refer to S1 Table.

## Results

### BEL Attenuates Nicotine-Induced Proliferation and Migration of Cultured Breast Cancer Cells

**Fig 1:**
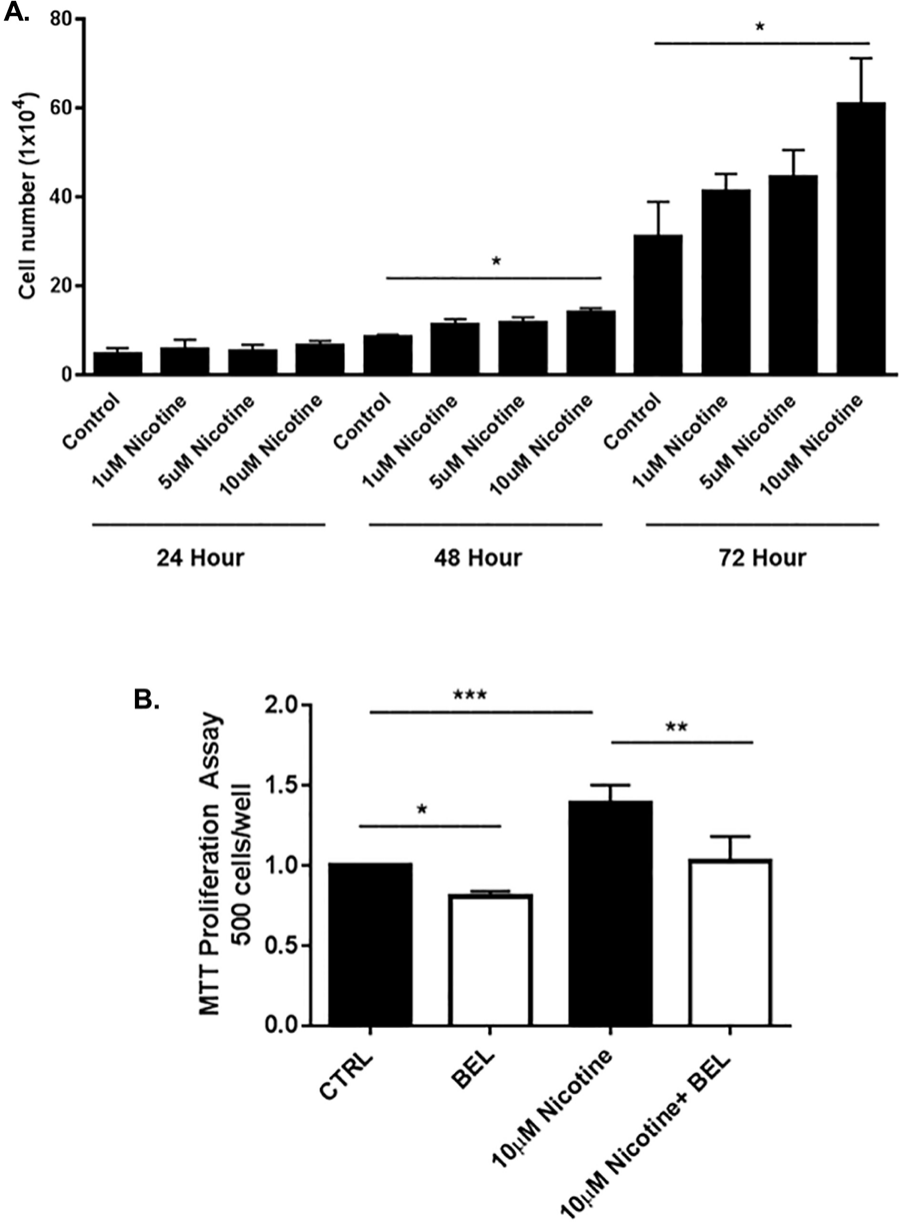
Nicotine-induced Cell Proliferation is attenuated by BEL. Promotion of 4T1 cell proliferation by nicotine dose dependently (1, 5, 10μM) was analyzed by cell count analysis for time intervals indicated above. Control wells were treated with DMSO (**A).** Cells were treated with nicotine (10μM) in the presence or absence of BEL (3μM) and proliferation measured by MTT assay **(B).** (n = 6–7). *, p<0.05 **, p<0.01 ***, p<0.00; two and one-way ANOVA.

To examine the effects of nicotine on cell growth, 4T1 breast cancer cells were treated with varying concentrations of nicotine (1, 5, 10 μM). At both 48 and 24 hours nicotine 10μM was found to significantly exacerbate breast cancer cell proliferation (Fig 1A); the use of this dose in cell culture has been previously documented [9]. Additionally, an MTT assay found BEL significantly attenuated both basal and nicotine-induced breast cancer proliferation (Fig 1B).

**Fig 2:**
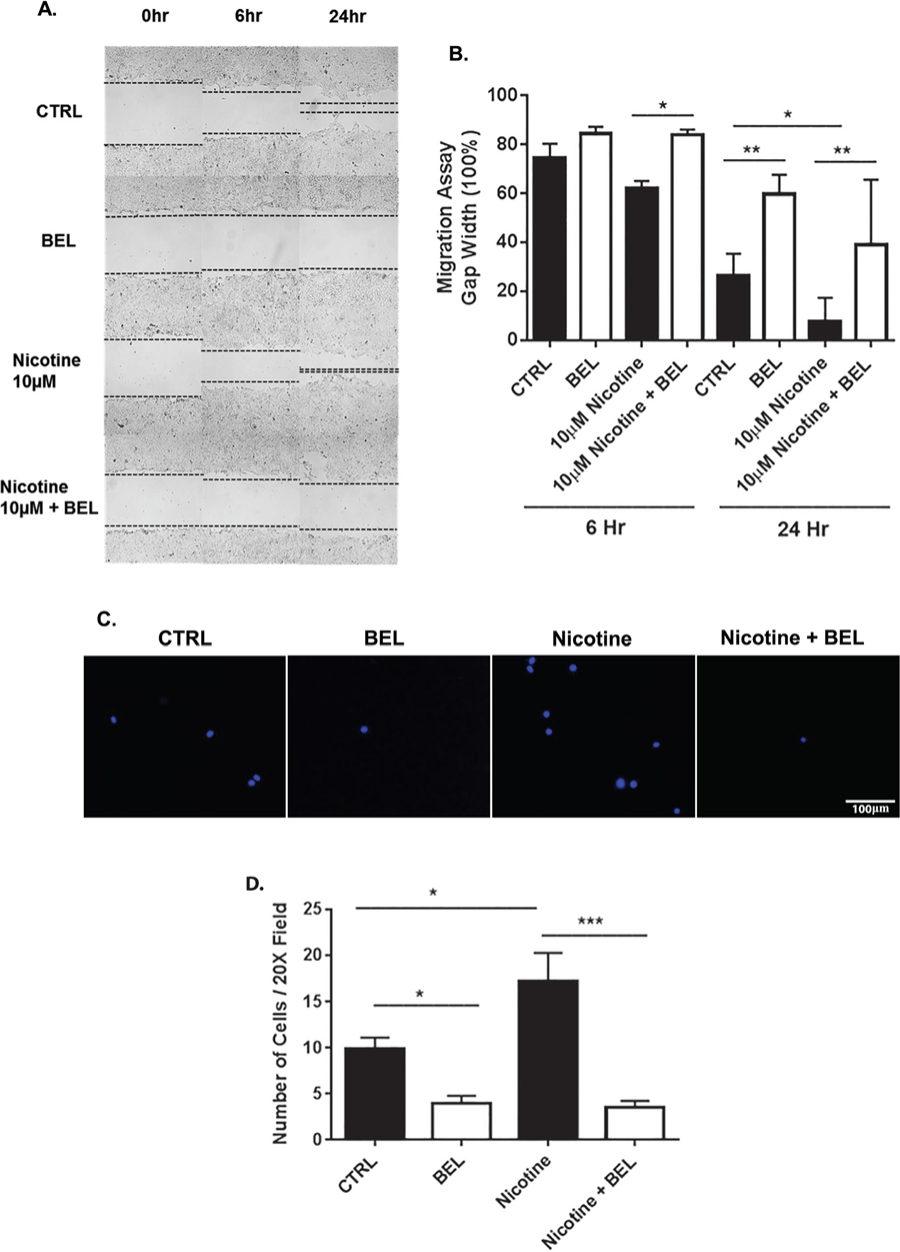
Nicotine-induced Cell Migration is decreased by BEL. Cultured 4T1 cells were scratched under normal conditions (DMSO) and nicotine (10μM) in the presence or absence of BEL (3μM). Representative 10x images of scratch migration assay, indicating BEL treatment attenuated nicotine induced migration (**A)**. Cell migration was quantitatively evaluated by measuring the distance between the scratch edges after 6, 24, and 48 hr **(B).** Representative images of Boyden chamber migration assay, cells were treated with nicotine (10M) in the presence or absence of BEL (3μM, 30 minute pretreatment) for 4 hr **(C).** Cell migration was quantitatively evaluated by counting the number of cells migrated **(D).** (n = 3–5). *, p<0.05 **,p<0.01; one-way ANOVA.

Furthermore, BEL was found to attenuate nicotine-induced breast cancer cell migration. Specifically BEL was found to attenuate gap closure promoted by nicotine at 6 and 24 hours (Fig 2A and 2B). The rate of gap closure can be facilitated by migration mechanisms and proliferation, hence, a short time point of 6 hours was investigated to show a reduction in cell migration ability. To further verify BEL attenuates nicotine-induced breast cancer migration a transwell assay was assessed at 4 hours (Fig 2C and 2D). Interestingly, for both migration experiments BEL was found to reduce basal breast cancer cell migration. Taken together this data suggests that iPLA_2_β is a main regulator of both basal and nicotine-induced breast cancer cell proliferation and migration.

### BEL Decreases Nicotine-induced MMP-9 Expression and Secretion

**Fig 3:**
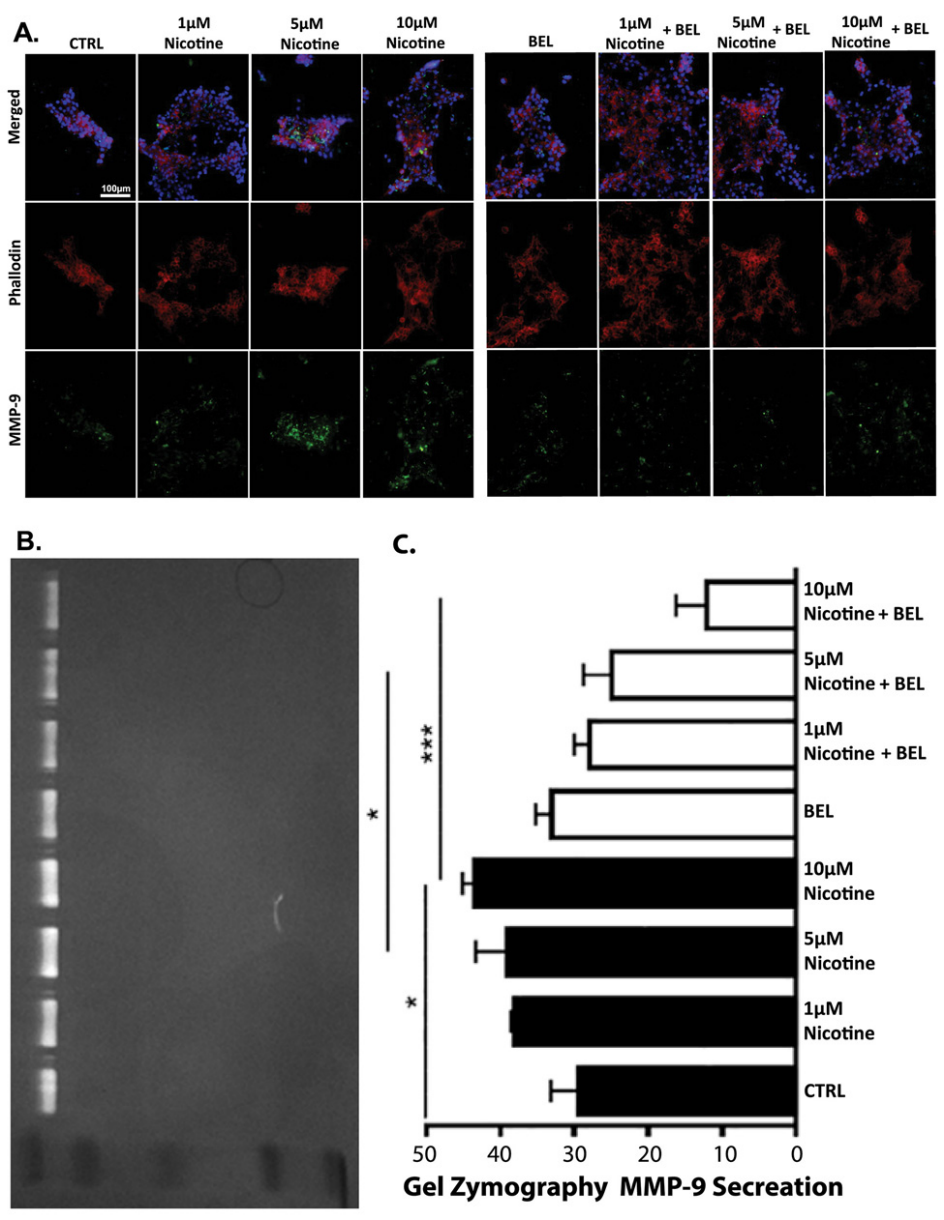
BEL decreases Nicotine-induced MMP-9 Expression and Secretion. An In-situ zymography was conducted with cells treated dose dependently with nicotine (1, 5, 10μM) in the presence or absence of BEL (3μM) for 24 hr. Cells were stained with MMP-9, phalloidin, and DAPI **(A).** Cell culture media was isolated from cells treated as previously described and a gel zymography was performed; the gel molecular weight standard from top to bottom is 103kDa, 77kDa, 49kDa, 34kDa, and 28kDa. A representative image of gel zymography shows a band corresponding to the molecular weight of proMMP-9 (92kDa) **(B)** and the area of gelatin dissolved was quantified (**C)**. n = 3, *,p<0.05. ***, p<0.001; one-way ANOVA.

iPLA_2_β regulation of MMPs in breast cancer cells was investigated to determine if the role iPLA_2_β could lead to a substantial impact on overall metastasis progression. To identify which MMP is secreted by the Nicotine stimulation, a series of parallel gel zymography experiments were performed. Our findings suggests nicotine increases MMP-9 expression (Fig 3A) and secretion (Fig 3B and 3C), which is attenuated by BEL. Subsequently, MMP-2 was not found to be highly secreted by basal breast cancer cells or during nicotine stimulation which is consistent with others showing a major pro-MMP-9 band, the molecular weight around 92kDa [50]. Interestingly, BEL showed a significant attenuation of basal MMP-9 expression but not secretion (Fig 3A, 3B and 3C).

**Fig 4:**
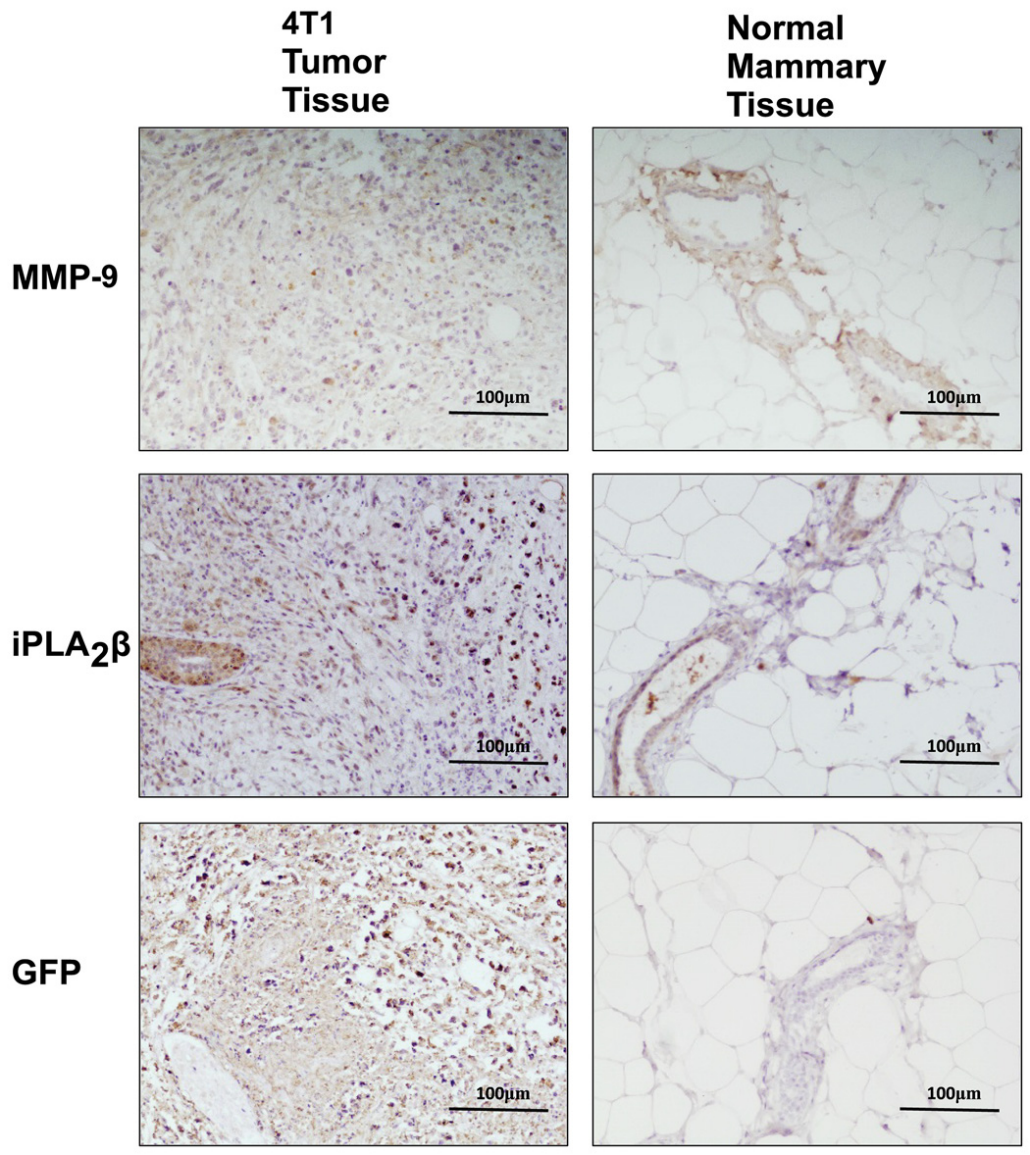
iPLA_2_β and MMP-9 Expression is upregulated in 4T1 tumor tissue. Normal mammary or 4T1 tumor tissue grown for 2wks in 12wk old Female BALB/c mice were isolated and embedded in paraffin. Tissue sections were stained with anti-GFP (green-fluorescent protein), iPLA_2_β, or MMP-9 antibodies. Positive staining is indicated by dark brown coloration and representative images shown.

Furthermore, in breast tumor tissue iPLA_2_β was found overexpressed compared to normal mammary tissue (Fig 4). Subsequently, MMP-9 was found overexpressed in the tumor tissue (identified by GFP staining) and not MMP-2 (data not shown). This data provides strong evidence that iPLA_2_β may promote MMP-9 production and expression in breast cancer tumor tissue and suggests that iPLA_2_β may be a major mediator of metastasis in highly invasive stage IV breast cancer.

### BEL Attenuates Nicotine-Induced Breast Cancer Tumor Growth

**Fig 5:**
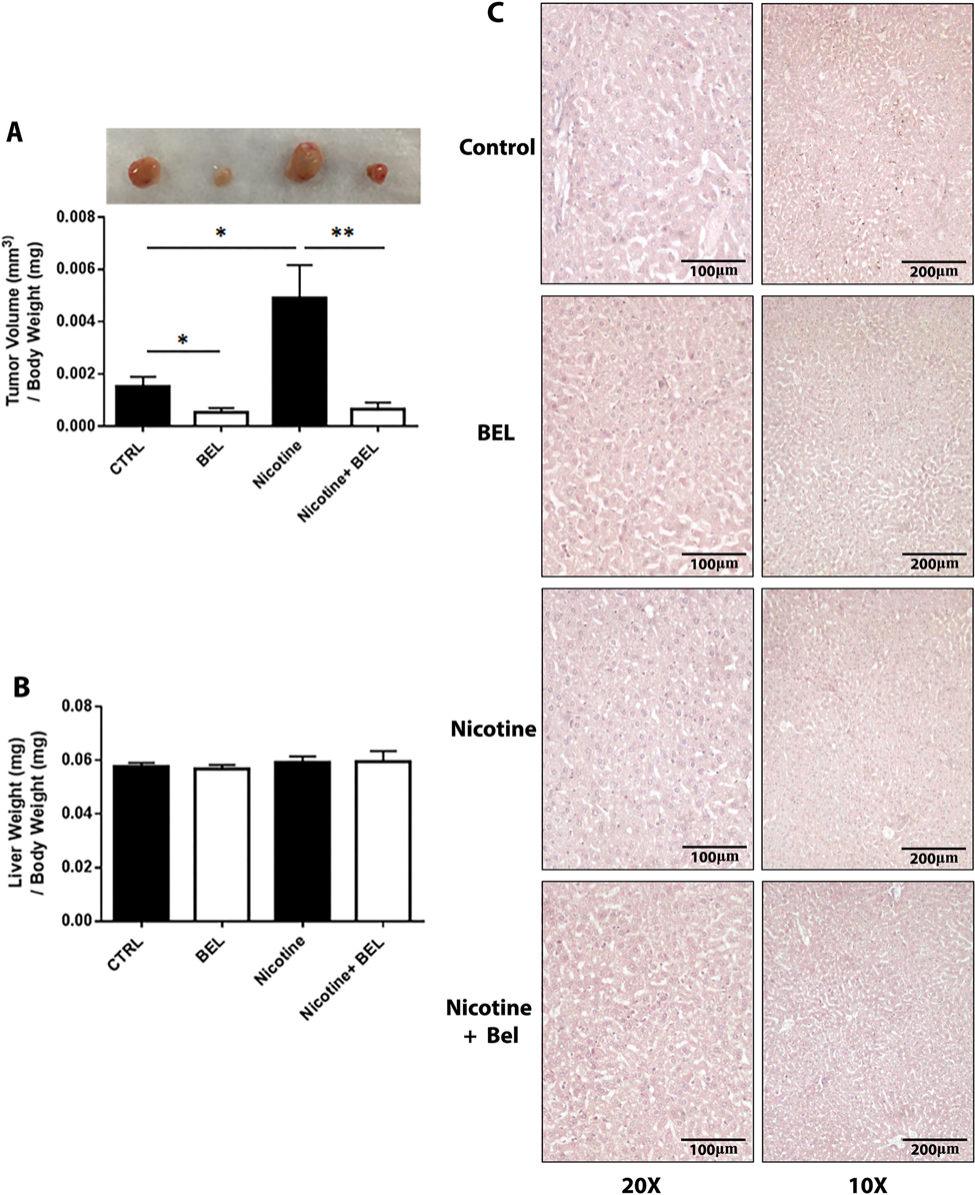
Nicotine-induced Tumor Growth is decreased by BEL. Female BALB/c mice were implanted with 4T1 cells (1X10^5^) in the right second mammary fat pad. Tumors were grown for 2wks in mice infused either with nicotine (5mg/kg/day) or saline (50% DMSO) and in the presence or absence of BEL (10ug/g/day). Tumors were isolated and final tumor volume **(A)** and liver weight **(B)** measured. Liver cytotoxicity was analyzed by HE staining with representative 10x and 20x images shown **(C)** (n = 8). *, p<0.05 **, p<0.01; unpaired t-test.

To examine the role of iPLA_2_β on nicotine-induced tumor growth, we administered BEL (10ug/g/day) to mice with tumors grown in the presence or absence of nicotine (5mg/kg/day). This dose of BEL is indicated to attenuate ovarian cancer development through reducing the adhesion, migration and invasion of epithelial ovarian cancer cells [51]. Subsequently, delivery of 5mg/kg/day of nicotine has been reported as an appropriate dose and is consistent with physiological range of blood nicotine levels in regular smokers [44,52]. Our data suggests that BEL significantly attenuated nicotine-induced tumor volume, along with decreasing normal tumor growth (Fig 5A). A decrease in basal tumor growth by BEL treatment was also found. Additionally, no physiological side effects from BEL were visible, subsequently, was no alteration in liver weight (Fig 5B) or liver cytotoxicity (Fig 5C).

### Nicotine-Induced Metastatic Tumor Environment and Progression Is Decreased by BEL

**Fig 6:**
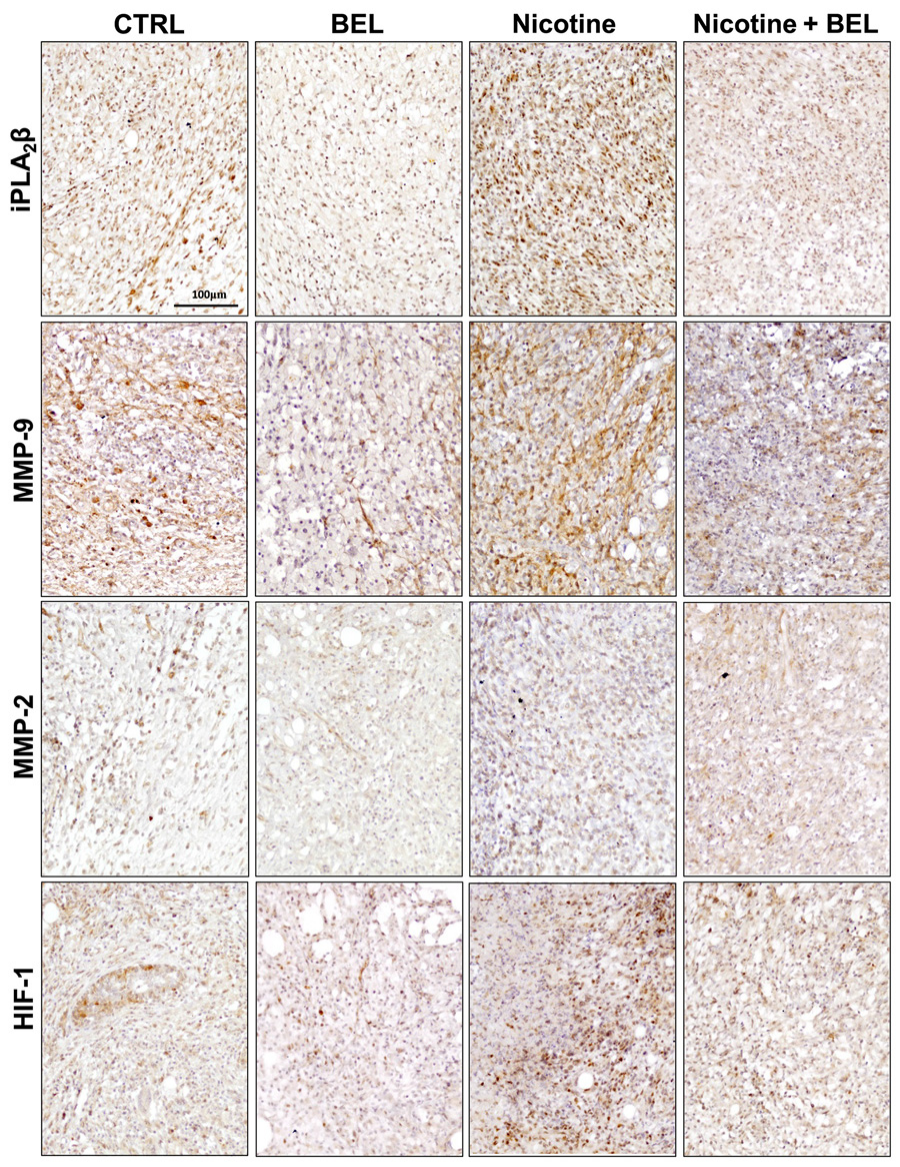
Nicotine-induced iPLA_2_β and MMP-9 Expression in Tumor Tissue is decreased by BEL. Control versus BEL and Nicotine versus Nicotine plus BE (n = 4): 4T1 tumor tissue grown for 2wks in 12wk old Female BALB/c mice were isolated and embedded in paraffin. Tissue sections were stained with anti-iPLA_2_β, MMP-9, MMP-2, and HIF-1alpha antibodies. Positive staining is indicated by dark brown coloration and representative images shown.

To determine the ability of nicotine to promote breast cancer metastasis, tissue immunohistochemistry was utilized. Nicotine was found to upregulate iPLA_2_β expression in the tumor tissue, which was attenuated by BEL along with basal iPLA_2_β expression (Fig 6). Subsequently, to elucidate the mechanism via which iPLA_2_β enhances nicotine-induced breast cancer metastasis we investigated MMP expression. We found that nicotine enhanced MMP-9 expression in the tumor tissue and both nicotine-induced and basal MMP-9 expression was decreased by BEL (Fig 6). Additionally, no difference was found in MMP-2 expression during nicotine or BEL treatment (Fig 6), representative images from four mice groups were imaged (S1 Fig). This data suggests that nicotine could promote metastasis through upregulating MMP-9 in an iPLA_2_β dependent manner.

**Fig 7:**
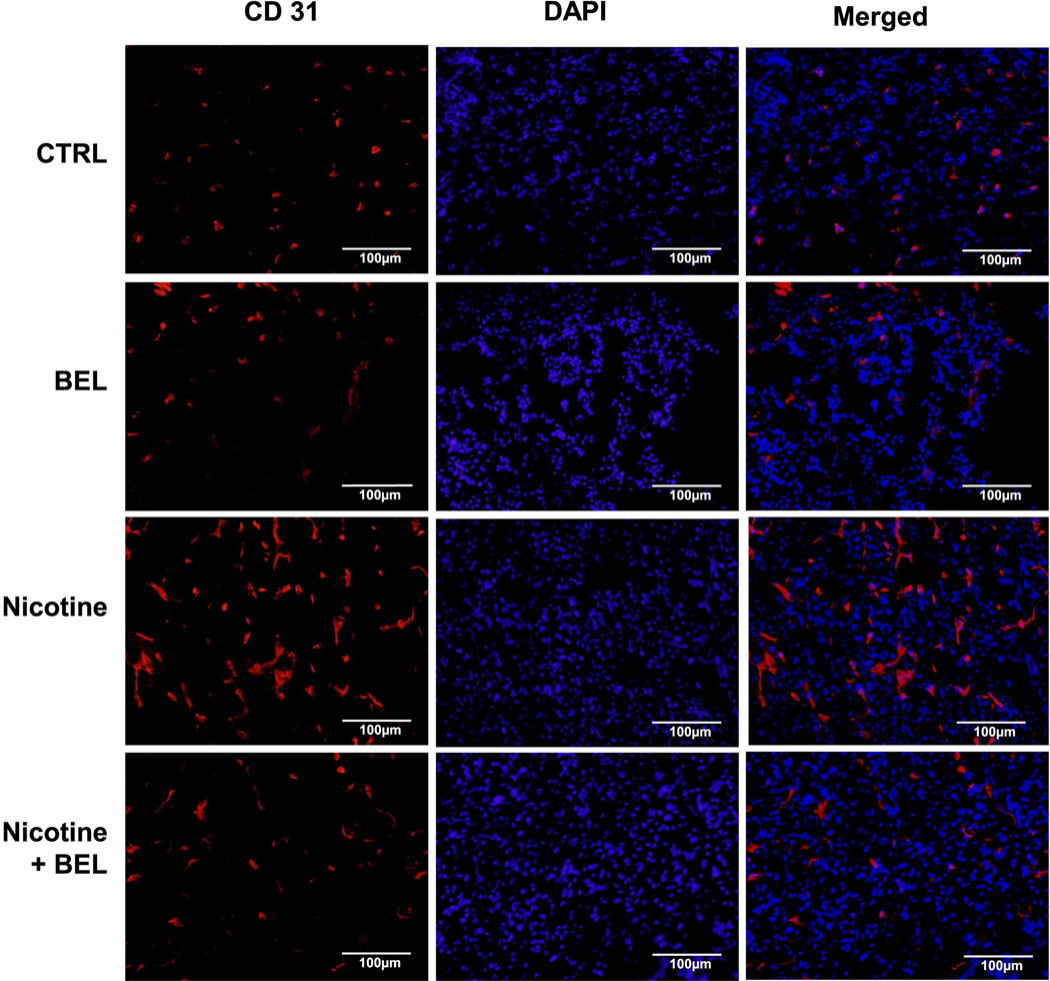
Nicotine-induced CD31 is decreased by BEL. Isolated tumor tissue was embedded in OCT and stained with CD31 and counterstained with DAPI. Representative images are shown. n = 4.

Furthermore, tumor angiogenesis plays a major role in tumor cell intravasation and metastasis. Our preliminary data suggests that nicotine increases both HIF-1alpha (Fig 6) and CD31 expression (Fig 7) in breast cancer tumor tissue, which is attenuated by BEL treatment. Hypoxic tumor tissue produces an environment which promotes angiolytic factor production and blood vessel formation. In Fig 6 we found that nicotine exacerbates tumor hypoxia conditions as an increase in HIF-1alpha was found. Subsequently, there was an increase in endothelial cells in the nicotine-induced tumor tissue, suggesting an increase in blood vessel presence and formation within the tumor tissue. This suggests that nicotine increase the accessibility of the breast cancer cells to blood vessels, hence, facilitating metastasis.

**Fig 8:**
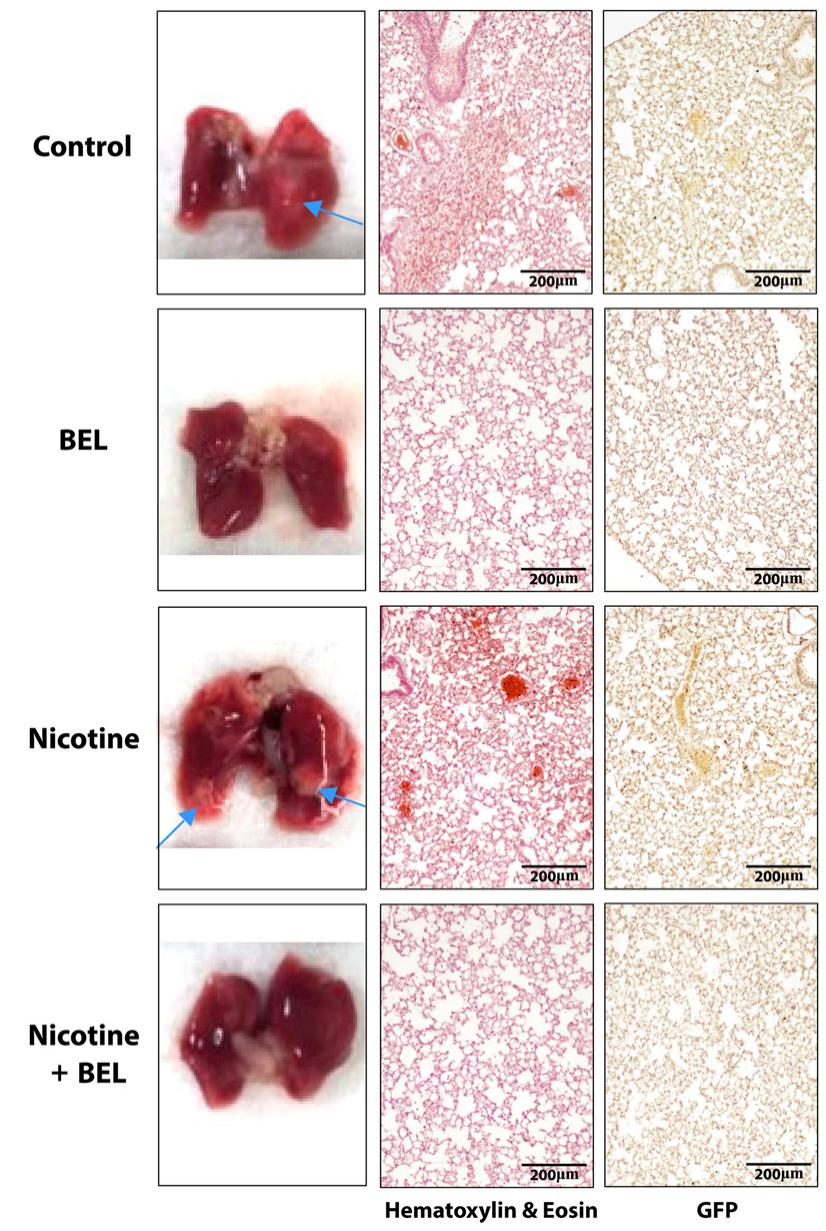
Nicotine-induced breast cancer metastasis in lung tissue was attenuated by BEL. Lungs were isolated and representative 10x images of tumor colonization shown by HE and GFP staining.

**Fig 9:**
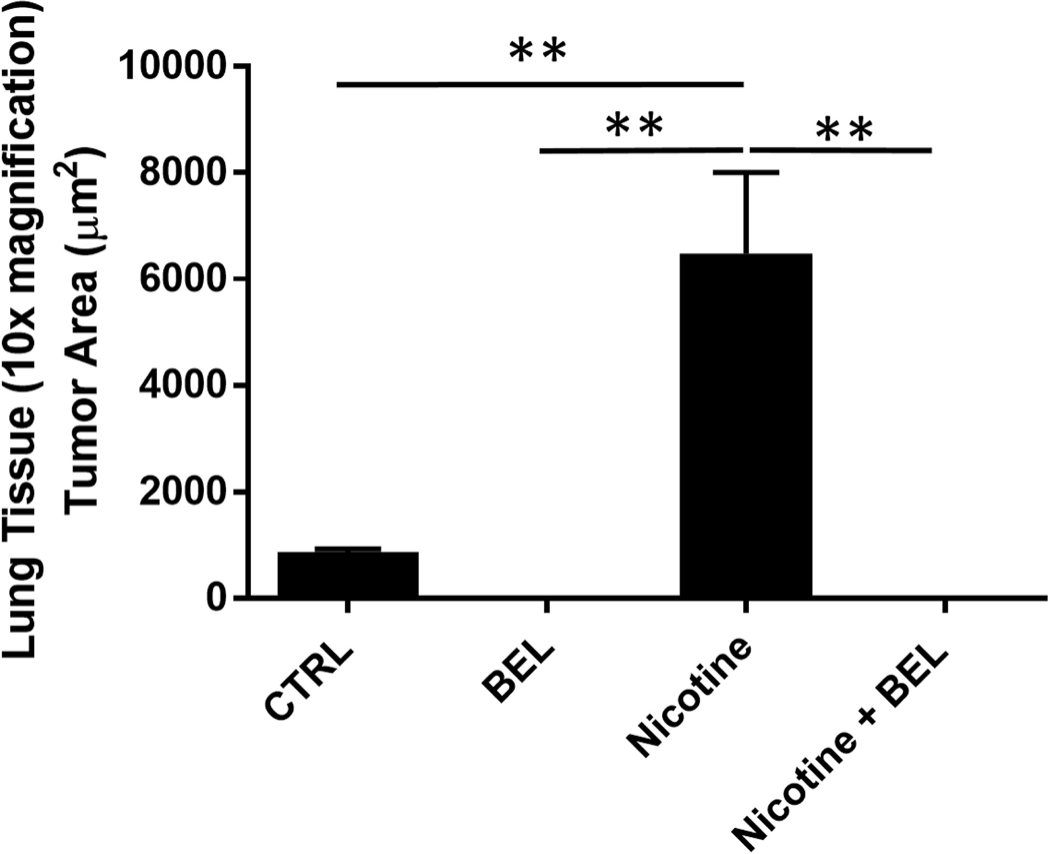
Nicotine-induced breast cancer metastasis in lung tissue was attenuated by BEL. Tumor area (μm^2^) was quantified in the lung sections stained with HE. n = 4, **, p<0.01; one-way ANOVA.

From our investigation of the tumor tissue we found that nicotine induces an environment highly suitable for promoting metastasis. To further examine metastasis we isolated the lung tissue and found a visible increase in breast cancer tumor colonization within the lungs from mice treated with nicotine (Fig 8). Moreover, BEL treatment decreased both nicotine-induced and basal lung metastasis. Subsequently, lung tissue sections stained with HE and GFP showed nicotine-induced tumor colonization was attenuated by BEL treatment (Fig 8). Area analysis conduction on the HE stained lung sections showed nicotine significantly enhanced tumor colonization in the lung tissue which was attenuated by BEL (Fig 9).

## Discussion

The exact cellular mechanisms regulating nicotine-induced breast cancer development remain unclear. However, cigarette smoke has been shown to enhance breast cancer cell mobility, along with, metastasis and adherence to lung endothelium in an iPLA_2_β dependent manner [7,53,54]. Recently, iPLA_2_β has emerged as a main regulator of cancer development and progression including prostate cancer cell growth, lung cancer cell invasion, and ovarian cancer cell proliferation [21,22] [23,55,56]. More importantly, iPLA_2_β has been shown to mediate cigarette smoke-induced cell proliferation and migration. Subsequently, our study provides the first research indicating that nicotine-induced breast cancer cell proliferation and migration occurs in an iPLA_2_β dependent manner.

The data presented in this study lays a preliminary foundation that targeting iPLA_2_β is a promising new strategy against nicotine-induced stage IV metastatic breast cancer. Inhibition of iPLA_2_β with BEL strongly attenuated nicotine-induced breast cancer proliferation and migration *in-vitro* and showed a decrease in both tumor growth and metastasis *in -vivo*. In women, adverse outcome from metastatic breast cancer is the leading cause of death among cancer cases, suggesting the critical need for novel pharmacological strategies to maintain and combat tumor progression and metastasis.

Previous work indicates that breast cancer tumors grown in genetically suppressed iPLA_2_β knockout mice showed no reduction in tumor size, however, an 11-fold attenuation in lung metastasis was found [53]. Importantly, our study shows that iPLA_2_β expression is upregulated in 4T1 tumors compared to normal mammary tissue, and furthermore, direct intraperitoneal administration of BEL attenuated both control and nicotine-induced tumor size and metastasis. This leads to an important observation that inhibition of iPLA_2_β in the tumors environment alone is not sufficient to influence tumor size and that direct suppression of iPLA_2_β in the cancer cells is needed to attenuate tumor development along with metastasis.

Additionally, our data suggests BEL is an effective treatment for early-stage breast cancer as it will interfere with the critical steps of cellular proliferation, migration, and metastasis. Furthermore, our study found a significant effect of BEL on short term exposure of nicotine on aggressive stage IV 4T1 cells. This provides evidence along with others that BEL will be effective during long term exposure in patients and potentially in less invasive breast cancer cells lines [7]. Moreover, studies using low doses of BEL in mice show toxicity tolerance and no liver toxicity as was similarly found in our study [51,57]. Additionally, supporting the limited toxicity and side effects that would occur in women from BEL treatments, is our results showing increase iPLA_2_β expression in the tumor tissue, while others have found that iPLA_2_β is not an essential gene in normal tissue in female mice [58]. Pharmacological drug design is progressing into tumor-specific treatments; in which BEL should be considered. To determine the mechanism promoting nicotine-induced breast cancer migration and metastasis, both MMP-2 and MMP-9 production and secretion were assessed. The production of MMP-2 and MMP-9 are considered by many publications as tumor markers, in which, they promote the development and progression of tumor cells, along with facilitating migration, invasion and metastasis [59]. High levels of MMP-9 are known to promote breast cancer invasion and migration to distant tissue sites [59] and downregulation of MMP-9 has been shown to be an effective mechanism to attenuate metastasis [60]. Furthermore, overexpression of MMP-9 in breast cancer has been shown to facilitate tumor cell invasiveness [61]. Our results supports the role that iPLA_2_β regulates 4T1 breast cancer cell production of MMP-9, which is amplified by nicotine. Moreover, BEL significantly reduced MMP-9 production, secretion and sequentially, lead to a decrease in both basal and nicotine-induced metastasis and colonization in lung tissue.

### Future Directions

The nicotinic acetylcholine receptors (nAChR) form either heteropentamers, consisting of a combination of α (α1-α6) and β (β2-β4) subunits or homopentamers derived from α7-α10 subunits [41]. To date, analysis of nAchRs expressed on mouse 4T1 breast cancer cells has not been thoroughly conducted. However, the expression of nAchRs on various human breast cancer cell lines have been examined including α9-nAChR and α7-nAChR which are found to promote human breast cancer cell proliferation and invasion [41,45,62]. Further experiments investigating the expression levels of the nAChR subtypes on 4T1 cells should be conducted along with determining with the nAChR potentially involved with nicotine-induced 4T1 cell proliferation and migration. Subsequently, further research needs to be conducted to determine the exact cellular role of iPLA_2_β in mediating both basal and nicotine-induced MMP-9 expression and secretion *in-vivo*.

### Conclusion

Taken together our research suggests that nicotine exacerbates the severity of breast cancer through facilitating metastasis by enhancing MMP-9 production in an iPLA_2_β dependent manner. Furthermore, our study indicates that BEL treatment could attenuate nicotine-induced and (non-nicotine induced) breast cancer growth and metastasis.

## Supporting Information

S1 Fig MMP-2 staining of the primary tumor. Representative 20x images are shown from primary breast tumors stained with MMP-2. n = 4.
(EPS)

S1 Table Study Data. Observed experimental data analyzed in the results section and displayed in the manuscript’s figures.
(XLSX)

## References

[1] DeSantis CE, Lin CC, Mariotto AB, Siegel RL, Stein KD, Kramer JL, et al (2014) Cancer treatment and survivorship statistics, 2014. CA: a cancer journal for clinicians 64: 252–271. 24890451 10.3322/caac.21235

[2] Desantis C, Ma J, Bryan L, Jemal A (2013) Breast cancer statistics, 2013. CA: a cancer journal for clinicians. 10.3322/caac.21203 24114568

[3] Heyder C, Gloria-Maercker E, Hatzmann W, Niggemann B, Zanker KS, Dittmar T. (2005) Role of the beta1-integrin subunit in the adhesion, extravasation and migration of T24 human bladder carcinoma cells. Clinical & experimental metastasis 22: 99–106. 16086230 10.1007/s10585-005-4335-z

[4] Weigelt B, Peterse JL, van 't Veer LJ (2005) Breast cancer metastasis: markers and models. Nature reviews Cancer 5: 591–602. 16056258 10.1038/nrc1670

[5] van Zijl F, Krupitza G, Mikulits W (2011) Initial steps of metastasis: cell invasion and endothelial transmigration. Mutation research 728: 23–34. 10.1016/j.mrrev.2011.05.002 21605699 PMC4028085

[6] Murin S, Pinkerton KE, Hubbard NE, Erickson K (2004) The effect of cigarette smoke exposure on pulmonary metastatic disease in a murine model of metastatic breast cancer. Chest 125: 1467–1471. 15078760 10.1378/chest.125.4.1467

[7] Kispert S, Marentette J, McHowat J (2015) Cigarette smoke induces cell motility via platelet-activating factor accumulation in breast cancer cells: a potential mechanism for metastatic disease. Physiological reports 3. 10.14814/phy2.12318 PMC4393154 25802360

[8] Di Cello F, Flowers VL, Li H, Vecchio-Pagan B, Gordon B, Harbom K, et al (2013) Cigarette smoke induces epithelial to mesenchymal transition and increases the metastatic ability of breast cancer cells. Molecular cancer 12: 90 10.1186/1476-4598-12-90 23919753 PMC3750372

[9] Guha P, Bandyopadhyaya G, Polumuri SK, Chumsri S, Gade P, Kalvakolanu DV, et al (2014) Nicotine promotes apoptosis resistance of breast cancer cells and enrichment of side population cells with cancer stem cell-like properties via a signaling cascade involving galectin-3, alpha9 nicotinic acetylcholine receptor and STAT3. Breast cancer research and treatment 145: 5–22. 10.1007/s10549-014-2912-z 24668500 PMC4028025

[10] Bavarva JH, Tae H, Settlage RE, Garner HR (2013) Characterizing the Genetic Basis for Nicotine Induced Cancer Development: A Transcriptome Sequencing Study. PloS one 8: e67252 23825647 10.1371/journal.pone.0067252 PMC3688980

[11] Jayakumar R, Kanthimathi MS (2012) Dietary spices protect against hydrogen peroxide-induced DNA damage and inhibit nicotine-induced cancer cell migration. Food chemistry 134: 1580–1584. 10.1016/j.foodchem.2012.03.101 25005983

[12] Johnson KC, Miller AB, Collishaw NE, Palmer JR, Hammond SK, Salmon AG, et al (2011) Active smoking and secondhand smoke increase breast cancer risk: the report of the Canadian Expert Panel on Tobacco Smoke and Breast Cancer Risk (2009). Tobacco control 20: e2 10.1136/tc.2010.035931 21148114

[13] Luo J, Margolis KL, Wactawski-Wende J, Horn K, Messina C, Stefanick ML, et al (2011) Association of active and passive smoking with risk of breast cancer among postmenopausal women: a prospective cohort study. BMJ 342: d1016 10.1136/bmj.d1016 21363864 PMC3047002

[14] Nyante SJ, Gierach GL, Dallal CM, Freedman ND, Park Y, Danforth KN, et al (2014) Cigarette smoking and postmenopausal breast cancer risk in a prospective cohort. British journal of cancer 110: 2339–2347. 10.1038/bjc.2014.132 24642621 PMC4007228

[15] Pierce JP, Patterson RE, Senger CM, Flatt SW, Caan BJ, Natarajan L, et al (2014) Lifetime cigarette smoking and breast cancer prognosis in the After Breast Cancer Pooling Project. Journal of the National Cancer Institute 106: djt359 10.1093/jnci/djt359 24317179 PMC3906992

[16] Faraglia B, Chen SY, Gammon MD, Zhang Y, Teitelbaum SL, Neugut AI, et al (2003) Evaluation of 4-aminobiphenyl-DNA adducts in human breast cancer: the influence of tobacco smoke. Carcinogenesis 24: 719–725. 12727801 10.1093/carcin/bgg013

[17] Grando SA (2014) Connections of nicotine to cancer. Nature reviews Cancer 14: 419–429. 10.1038/nrc3725 24827506

[18] Talhout R, Schulz T, Florek E, van Benthem J, Wester P, Opperhuizen A. (2011) Hazardous compounds in tobacco smoke. International journal of environmental research and public health 8: 613–628. 10.3390/ijerph8020613 21556207 PMC3084482

[19] Nishioka T, Kim HS, Luo LY, Huang Y, Guo J, Chen CY. (2011) Sensitization of epithelial growth factor receptors by nicotine exposure to promote breast cancer cell growth. Breast cancer research: BCR 13: R113 10.1186/bcr3055 22085699 PMC3326555

[20] Momi N, Ponnusamy MP, Kaur S, Rachagani S, Kunigal SS, Chellappan S, et al (2013) Nicotine/cigarette smoke promotes metastasis of pancreatic cancer through alpha7nAChR-mediated MUC4 upregulation. Oncogene 32: 1384–1395. 10.1038/onc.2012.163 22614008 PMC3427417

[21] Nicotera TM, Schuster DP, Bourhim M, Chadha K, Klaich G, Corral DA. (2009) Regulation of PSA secretion and survival signaling by calcium-independent phopholipase A(2)beta in prostate cancer cells. The Prostate 69: 1270–1280. 10.1002/pros.20968 19475654

[22] Sun B, Zhang X, Yonz C, Cummings BS (2010) Inhibition of calcium-independent phospholipase A2 activates p38 MAPK signaling pathways during cytostasis in prostate cancer cells. Biochemical Pharmacology 79: 1727–1735. 10.1016/j.bcp.2010.02.005 20171194

[23] Song Y, Wilkins P, Hu W, Murthy KS, Chen J, Lee Z, et al (2007) Inhibition of calcium-independent phospholipase A2 suppresses proliferation and tumorigenicity of ovarian carcinoma cells. The Biochemical journal 406: 427–436. 10.1042/BJ20070631 PMC2049037 17555408

[24] Zhao X, Wang D, Zhao Z, Xiao Y, Sengupta S, Xiao Y, et al (2006) Caspase-3-dependent activation of calcium-independent phospholipase A2 enhances cell migration in non-apoptotic ovarian cancer cells. The Journal of biological chemistry 281: 29357–29368. 16882668 10.1074/jbc.M513105200

[25] Akiba S, Sato T (2004) Cellular function of calcium-independent phospholipase A2. Biological & pharmaceutical bulletin 27: 1174–1178. 15305016 10.1248/bpb.27.1174

[26] Balsinde J, Winstead MV, Dennis EA (2002) Phospholipase A(2) regulation of arachidonic acid mobilization. FEBS Lett 531: 2–6. 12401193 10.1016/s0014-5793(02)03413-0

[27] Balsinde J, Balboa MA (2005) Cellular regulation and proposed biological functions of group VIA calcium-independent phospholipase A2 in activated cells. Cell Signal 17: 1052–1062. 15993747 10.1016/j.cellsig.2005.03.002

[28] Ma Z, Turk J (2001) The molecular biology of the group VIA Ca2+-independent phospholipase A2. Prog Nucleic Acid Res Mol Biol 67: 1–33. 11525380 10.1016/s0079-6603(01)67023-5

[29] Calderon LE, Liu S, Su W, Xie Z, Guo Z, Eberhard W, et al (2012) iPLA2beta overexpression in smooth muscle exacerbates angiotensin II-induced hypertension and vascular remodeling. PloS one 7: e31850 10.1371/journal.pone.0031850 22363752 PMC3282780

[30] Xie Z, Gong MC, Su W, Xie D, Turk J, Guo Z. (2010) Role of calcium-independent phospholipase A2beta in high glucose-induced activation of RhoA, Rho kinase, and CPI-17 in cultured vascular smooth muscle cells and vascular smooth muscle hypercontractility in diabetic animals. The Journal of biological chemistry 285: 8628–8638. 10.1074/jbc.M109.057711 20086008 PMC2838285

[31] Ali T, Kokotos G, Magrioti V, Bone RN, Mobley JA, Hancock W, et al (2013) Characterization of FKGK18 as inhibitor of group VIA Ca2+-independent phospholipase A2 (iPLA2beta): candidate drug for preventing beta-cell apoptosis and diabetes. PloS one 8: e71748 10.1371/journal.pone.0071748 23977134 PMC3748103

[32] Liu S, Xie Z, Zhao Q, Pang H, Turk J, Calderon L, et al (2012) Smooth muscle-specific expression of calcium-independent phospholipase A2beta (iPLA2beta) participates in the initiation and early progression of vascular inflammation and neointima formation. The Journal of biological chemistry 287: 24739–24753. 10.1074/jbc.M112.340216 22637477 PMC3397901

[33] Vadali S, Post SR (2014) Lipid rafts couple class A scavenger receptors to phospholipase A2 activation during macrophage adhesion. Journal of leukocyte biology 96: 873–881. 10.1189/jlb.2A0414-214R 25070949 PMC4197562

[34] Nikolic DM, Gong MC, Turk J, Post SR (2007) Class A scavenger receptor-mediated macrophage adhesion requires coupling of calcium-independent phospholipase A(2) and 12/15-lipoxygenase to Rac and Cdc42 activation. The Journal of biological chemistry 282: 33405–33411. 17873277 10.1074/jbc.M704133200 PMC2080787

[35] Ackermann EJ, Conde-Frieboes K, Dennis EA (1995) Inhibition of macrophage Ca(2+)-independent phospholipase A2 by bromoenol lactone and trifluoromethyl ketones. J Biol Chem 270: 445–450. 7814408 10.1074/jbc.270.1.445

[36] Hazen SL, Zupan LA, Weiss RH, Getman DP, Gross RW (1991) Suicide inhibition of canine myocardial cytosolic calcium-independent phospholipase A2. Mechanism-based discrimination between calcium-dependent and -independent phospholipases A2. J Biol Chem 266: 7227–7232. 2016324

[37] Pulaski BA, Ostrand-Rosenberg S (2001) Mouse 4T1 breast tumor model. Current protocols in immunology / edited by Coligan John E [et al] Chapter 20: Unit 20 22. 10.1002/0471142735.im2002s39 18432775

[38] Benowitz NL, Hukkanen J, Jacob P 3rd (2009) Nicotine chemistry, metabolism, kinetics and biomarkers. Handbook of experimental pharmacology: 29–60. 10.1007/978-3-540-69248-5_2 19184645 PMC2953858

[39] Andersson G, Vala EK, Curvall M (1997) The influence of cigarette consumption and smoking machine yields of tar and nicotine on the nicotine uptake and oral mucosal lesions in smokers. Journal of oral pathology & medicine: official publication of the International Association of Oral Pathologists and the American Academy of Oral Pathology 26: 117–123. 10.1111/j.1600-0714.1997.tb00033.x 9083935

[40] Hao J, Shi FD, Abdelwahab M, Shi SX, Simard A, Whiteaker P, et al (2013) Nicotinic receptor beta2 determines NK cell-dependent metastasis in a murine model of metastatic lung cancer. PloS one 8: e57495 10.1371/journal.pone.0057495 23469004 PMC3585320

[41] Chen CS, Lee CH, Hsieh CD, Ho CT, Pan MH, Huang CS, et al (2011) Nicotine-induced human breast cancer cell proliferation attenuated by garcinol through down-regulation of the nicotinic receptor and cyclin D3 proteins. Breast cancer research and treatment 125: 73–87. 10.1007/s10549-010-0821-3 20229177

[42] Wilkinson DS, Turner JR, Blendy JA, Gould TJ (2013) Genetic background influences the effects of withdrawal from chronic nicotine on learning and high-affinity nicotinic acetylcholine receptor binding in the dorsal and ventral hippocampus. Psychopharmacology 225: 201–208. 10.1007/s00213-012-2808-8 22836371 PMC3755015

[43] Wang S, Zhang C, Zhang M, Liang B, Zhu H, Lee J, et al (2012) Activation of AMP-activated protein kinase alpha2 by nicotine instigates formation of abdominal aortic aneurysms in mice in vivo. Nature medicine 18: 902–910. 10.1038/nm.2711 22561688 PMC3559018

[44] Davis JA, James JR, Siegel SJ, Gould TJ (2005) Withdrawal from chronic nicotine administration impairs contextual fear conditioning in C57BL/6 mice. The Journal of neuroscience: the official journal of the Society for Neuroscience 25: 8708–8713. 16177040 10.1523/JNEUROSCI.2853-05.2005 PMC2697573

[45] Lee CH, Chang YC, Chen CS, Tu SH, Wang YJ, Chen LC, et al (2011) Crosstalk between nicotine and estrogen-induced estrogen receptor activation induces alpha9-nicotinic acetylcholine receptor expression in human breast cancer cells. Breast cancer research and treatment 129: 331–345. 10.1007/s10549-010-1209-0 20953833

[46] Liu Z, Zhang B, Liu K, Ding Z, Hu X (2012) Schisandrin B attenuates cancer invasion and metastasis via inhibiting epithelial-mesenchymal transition. PloS one 7: e40480 10.1371/journal.pone.0040480 22848381 PMC3405072

[47] Kanaya N, Adams L, Takasaki A, Chen S (2014) Whole blueberry powder inhibits metastasis of triple negative breast cancer in a xenograft mouse model through modulation of inflammatory cytokines. Nutrition and cancer 66: 242–248. 10.1080/01635581.2014.863366 24364759

[48] Mandal CC, Ghosh-Choudhury T, Yoneda T, Choudhury GG, Ghosh-Choudhury N (2010) Fish oil prevents breast cancer cell metastasis to bone. Biochemical and biophysical research communications 402: 602–607. 10.1016/j.bbrc.2010.10.063 20971068 PMC2993881

[49] Liu S, Xie Z, Daugherty A, Cassis LA, Pearson KJ, Gong MC, et al (2013) Mineralocorticoid receptor agonists induce mouse aortic aneurysm formation and rupture in the presence of high salt. Arteriosclerosis, thrombosis, and vascular biology 33: 1568–1579. 10.1161/ATVBAHA.112.300820 23661677 PMC3707291

[50] Ramos-DeSimone N, Hahn-Dantona E, Sipley J, Nagase H, French DL, Quigley JP. (1999) Activation of matrix metalloproteinase-9 (MMP-9) via a converging plasmin/stromelysin-1 cascade enhances tumor cell invasion. The Journal of biological chemistry 274: 13066–13076. 10224058 10.1074/jbc.274.19.13066

[51] Li H, Zhao Z, Antalis C, Zhao Z, Emerson R, Wei G, et al (2011) Combination therapy of an inhibitor of group VIA phospholipase A2 with paclitaxel is highly effective in blocking ovarian cancer development. The American journal of pathology 179: 452–461. 10.1016/j.ajpath.2011.03.027 21703423 PMC3123860

[52] Wang Q, Zhang M, Liang B, Shirwany N, Zhu Y, Zou MH. (2011) Activation of AMP-activated protein kinase is required for berberine-induced reduction of atherosclerosis in mice: the role of uncoupling protein 2. PloS one 6: e25436 10.1371/journal.pone.0025436 21980456 PMC3181327

[53] McHowat J, Gullickson G, Hoover RG, Sharma J, Turk J, Kornbluth J. (2011) Platelet-activating factor and metastasis: calcium-independent phospholipase A2beta deficiency protects against breast cancer metastasis to the lung. American journal of physiology Cell physiology 300: C825–832. 10.1152/ajpcell.00502.2010 21228317 PMC3074634

[54] Kispert SE, Marentette JO, McHowat J (2014) Enhanced breast cancer cell adherence to the lung endothelium via PAF acetylhydrolase inhibition: a potential mechanism for enhanced metastasis in smokers. American journal of physiology Cell physiology 307: C951–956. 10.1152/ajpcell.00218.2014 25186013 PMC4233261

[55] Ho JN, Lee SB, Lee SS, Yoon SH, Kang GY, Hwang SG, et al (2010) Phospholipase A2 activity of peroxiredoxin 6 promotes invasion and metastasis of lung cancer cells. Molecular cancer therapeutics 9: 825–832. 10.1158/1535-7163.MCT-09-0904 20354123

[56] Jo M, Yun HM, Park KR, Hee Park M, Myoung Kim T, Ho Pak J, et al (2013) Lung tumor growth-promoting function of peroxiredoxin 6. Free radical biology & medicine 61C: 453–463. 10.1016/j.freeradbiomed.2013.04.032 23643677

[57] Saab-Aoude S, Bron AM, Creuzot-Garcher CP, Bretillon L, Acar N (2013) A mouse model of in vivo chemical inhibition of retinal calcium-independent phospholipase A2 (iPLA2). Biochimie 95: 903–911. 10.1016/j.biochi.2012.12.008 23266358

[58] Bao S, Miller DJ, Ma Z, Wohltmann M, Eng G, Ramanadham S, et al (2004) Male mice that do not express group VIA phospholipase A2 produce spermatozoa with impaired motility and have greatly reduced fertility. The Journal of biological chemistry 279: 38194–38200. 15252026 10.1074/jbc.M406489200 PMC3733543

[59] Radenkovic S, Konjevic G, Jurisic V, Karadzic K, Nikitovic M, Gopcevic K. (2013) Values of MMP-2 and MMP-9 in Tumor Tissue of Basal-Like Breast Cancer Patients. Cell biochemistry and biophysics. 10.1007/s12013-013-9701-x 23812723

[60] Lee SJ, Hong S, Yoo SH, Kim GW (2013) Cyanidin-3-O-Sambubioside from Acanthopanax sessiliflorus Fruit Inhibits Metastasis by Downregulating MMP-9 in Breast Cancer Cells MDA-MB-231. Planta medica. 10.1055/s-0033-1350954 24214832

[61] Choi JY, Jang YS, Min SY, Song JY (2011) Overexpression of MMP-9 and HIF-1alpha in Breast Cancer Cells under Hypoxic Conditions. Journal of breast cancer 14: 88–95. 10.4048/jbc.2011.14.2.88 21847402 PMC3148536

[62] Dasgupta P, Rizwani W, Pillai S, Kinkade R, Kovacs M, Rastogi S, et al (2009) Nicotine induces cell proliferation, invasion and epithelial-mesenchymal transition in a variety of human cancer cell lines. International journal of cancer Journal international du cancer 124: 36–45. 10.1002/ijc.23894 18844224 PMC2826200

